# Impact of histotypes on preferential organ‐specific metastasis in triple‐negative breast cancer

**DOI:** 10.1002/cam4.2759

**Published:** 2019-12-09

**Authors:** Yaming Li, Peng Su, Yifei Wang, Hanwen Zhang, Yiran Liang, Ning Zhang, Xiaojin Song, Xiaoyan Li, Jie Li, Qifeng Yang

**Affiliations:** ^1^ Department of Breast Surgery Qilu Hospital Shandong University School of Medicine Ji'nan Shandong China; ^2^ Department of Pathology Qilu Hospital of Shandong University Ji'nan Shandong China; ^3^ Department of Ultrasound Qilu Hospital of Shandong University Jinan China; ^4^ Pathology Tissue Bank Qilu Hospital Shandong University Jinan China

**Keywords:** histologic subtypes, preferential distant metastasis, prognosis, SEER, triple‐negative breast cancer

## Abstract

**Background:**

The distant metastasis was the most predictive characters of poor prognosis for triple‐negative breast cancer (TNBC). We aimed to evaluate the correlation between patient characters and preferential distant metastatic sites (DMS) and its effects on prognosis.

**Methods:**

Using the 2010‐2014 Surveillance, Epidemiology, and End Results Program (SEER) data, patients with TNBC were classified into eight histologic subtypes. Patient characters were compared using a chi‐squared test. Logistic regression was used for identification of predictive factors. The log‐rank testing was utilized with disease‐specific survival (DSS) and overall survival (OS) as the primary outcomes.

**Results:**

A total of 23 270 patients with TNBC were involved, including 1544 patients with distant metastatic cancer. Bone metastasis was diagnosed in 559 cases, brain metastasis in 124 cases, liver metastasis found in 369 cases and lung metastasis in 492 cases. Histologic subtypes including metaplastic breast carcinoma and invasive lobular carcinoma showed significant differences in preferential DMS compared with invasive ductal carcinoma. Furthermore, we found different histologic subtypes with specific DMS showed various prognosis. We also evaluated different DMS of specific histologic subtypes showed different prognosis.

**Conclusion:**

Certain histologic subtypes of breast cancer are associated with preferential DMS and prognosis; this knowledge may help to further understand the mechanism of breast cancer metastasis and to monitor the prognosis of patients with TNBC.

## Graphical abstract

This study was the first to explore the effects of patient characters on preferential distant metastasis sites in patients with triple‐negative breast cancer (TNBC). We proved specific histologic subtypes of TNBC could remarkable influence the preferential distant metastasis sites. We proved the prognoses of patients with TNBC varied among different histologic subtypes and when metastasized to specific organs.

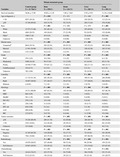

## 1 BACKGROUND

Breast cancer is one of the most common tumors among women, and the second leading cause of cancer‐related death in the world.^1^ Approximately 1 to 1.3 million cases are diagnosed with breast cancers worldwide every year, including approximately 60% patients with hormone receptor‐positive breast cancers, 20% patients with Her2/neu receptor overexpressed cancers, and triple‐negative breast cancers (TNBC) constitute approximately 20% of breast cancer cases.^2^

Traditionally, TNBC encompasses a subset of breast cancer that lacks the expression of estrogen receptor (ER), progesterone receptor (PR), and human epidermal growth factor receptor 2 (HER2), which requires special treatment approaches instead of endocrine therapy.^3^ Due to the ineffectiveness of current breast cancer–targeted therapies as well as more malignant behaviors, TNBC is associated with higher risk of distant recurrence, higher rates of metastases, higher probability of relapse, and worst overall survival (OS) compared to other subtypes.^4^ Previous reports showed that about 35% patients with TNBC were diagnosed with distant metastases within 5 years of initial diagnosis, and cases with progressive stage only have a median of 2‐year survival time.^5^, ^6^

The dissemination of breast cancer cells and eventual metastatic growth to distant organs, predominantly the bone, brain, lung, and liver, are the primary cause of death for the vast majority of patients with TNBC.^7^, ^8^ The distant metastasis is highly complex, yet poorly understood, and consists of multiple steps, in which the influencing and indicative factors have not been well evaluated. In fact, numerous studies have studied the mechanism of distant metastasis of TNBC.^9^, ^10^ For instance, the activation of CXCR4 receptor via its ligand CXCL12 or ANGPTL2 was found to induce MLK3 and Erk1/2 signaling and promote intravasation which leads to the development of lung and bone metastases.^9^ Zhuang et al reported that DKK1 promotes breast‐to‐bone metastasis by regulating canonical WNT signaling of osteoblasts and then suppressed lung metastasis.^10^ In another aspect, the pathomorphological indicators, as the results of genetic changes of tumor cells, should also be well studied. Previous study had reported that specific histologic subtypes showed significant differences in the percentage of distant metastasis, and invasive lobular carcinoma was considered as a subtype with highest metastasis probability.^11^ However, another study reported that breast cancer subtypes based on hormone receptor (HR) and human epidermal growth factor receptor 2 (HER2) status had specific preferential site of distant organ metastases.^12^ Surprisingly, despite TNBC was considered as the most invasive breast cancer subtype, no study has focused on effects of patients characters on the preferential site of distant organ metastases of TNBC.

In this study, we first evaluated the correlation between patient characters of TNBC and preferential distant metastatic sites based on a population‐based national registry. We also explored prognostic differences in subtypes within specific distant organs, and prognostic differences in specific subtype with different DMS. Our study broadens our knowledge on endogenous histologic heterogeneity of TNBC and guided individualized TNBC patient management in clinics.

## 2 METHODS

### 2.1 Data source and patient selection

We performed a retrospective cohort study using data from the Surveillance, Epidemiology and End Results (SEER) database. The SEER database currently collects data on patient demographics, tumor characteristics, first course of treatment, and follow‐up of vital status from 18 population‐based cancer registries, encompassing approximately 28% of the US population. Tumor histologic types are classified according to the International Classification of Diseases for Oncology (ICD‐O), 3rd edition. Tumor stage is categorized according to the American Joint Committee on Cancer (AJCC) staging system, 7th edition.

We identified potentially eligible patients based on the following inclusion criteria: female, aged between 18 and 85, years of diagnosis from 2010 to 2014, breast cancer as the first and only malignant cancer diagnosis, and TNBC. We excluded patients who lacked a histologically confirmed diagnosis and those identified by death certificate or autopsy.

We restricted our analysis to the eight most prevalent and well‐defined histologic types, that is, invasive ductal carcinoma (IDC, ICD‐O‐3 8500/3), metaplastic breast carcinoma (MBC, ICD‐O‐3 8560/3, 8570/3, 8571/3, 8572/3, 8575/3, and 8980/3), medullary breast carcinoma (MedBC, ICD‐O‐3 8510/3), mixed IDC and invasive lobular carcinoma (IDC‐ILC, ICD‐O‐3 8522/3), ILC (ICD‐O‐3 8520/3), apocrine carcinoma (ICD‐O‐3 8401/3), IDC mixed with other type (IDC‐other, ICD‐O‐3 8523/3), apocrine adenocarcinoma (AAC, ICD‐O‐3 8401/3), and inflammation breast cancer (IBC, ICD‐O‐3 8530/3). The remaining histologic types were not included in our analysis due to the small number of patients or the imprecise classification.

### 2.2 Statistical analysis

We compared the differences between nonmetastatic control patients and metastatic patients using the Pearson's chi‐squared test. The association of clinicopathologic factors with the sites of distant metastases was modeled with logistic regression analysis. Both univariate and multivariate odds ratios (ORs) and 95% confidence intervals (CIs) were calculated for each model. Predictive factors for distant metastasis were determined by multivariable logistic regression analysis, in which factors that were statistically significant in the univariate analysis were entered into the multivariable logistic regression analysis.

The survival differences between the groups were compared using the log‐rank test. Disease‐specific survival (DSS) was defined as the interval from the date of diagnosis to the date of death due to breast cancer. Overall survival was defined as the interval from the date of diagnosis to the date of death from any cause. The results were reported using hazard ratios (HRs) with 95% CIs.

Statistical analyses were performed using SPSS version 19.0 (SPSS, Inc). All tests were two‐sided, and the value of *P* < .05 was considered statistically significant.

## 3 RESULTS

### 3.1 Demographic, tumor, and treatment characteristics

The study groups consisted of a total of 23 270 patients with TNBC, including 1544 (6.64%) patients with clinically diagnosed distant metastasis. Among the 23 270 patients with TNBC, 20 638 (87.52%) patients were diagnosed with IDC, 708 (3.04%) patients with MBC, 303 (1.30%) with MedBC, 278 (1.19%) with ILC, 306 (1.31%) with IDC‐ILC, 657 (2.82%) with IDC‐oth, 211 (0.91%) with AAC, and 169 (0.73%) with IBC.

Table 1 outlines the demographic, tumor, and treatment characteristics of patients with TNBC according to metastasis status. In general, patients with distant metastasis have relative shorter survival time and were more likely to be older, unmarried, be found in paired or bilateral laterality, larger in sized, lymph node metastasis (each *P* < .05). Patients with lung metastasis showed comparative better prognosis while brain metastasis have shorter survival time among the four metastasis groups. Interestingly, we also found that Black patients were more likely to develop bone and lung metastasis. Furthermore, significant histologic differences were found among the control and distant metastasis groups. For instance, compared with the control group, the bone metastasis group showed fewer patients with IDC, MBC, IDC, and other groups showed more patients with LC, IDC, and LC, and IBC. Considering treatments for TNBC, we found that the radiotherapy acceptance rate by patients was significantly lower in the bone, liver, and lung metastasis groups, while patients with brain metastasis were more likely to accept radiotherapy. Moreover, the acceptance rate of chemotherapy by patients was also found to be lower in the lung metastasis group.

**Table 1:** Characteristics of patients with TNBC from the SEER database by metastasis patterns

Variables	Control group N = 21 726(%)	Distant metastasis group			
		Bone N = 559(%)	Brain N = 124 (%)	Liver N = 369 (%)	Lung N = 492 (%)
Survival (months)	26.66 ± 17.09	10.94 ± 11.30	7.68 ± 7.642	9.70 ± 9.93	11.19 ± 10.60
Age at diagnosis, y		***P* = .010**	*P* = .231	*P* = .440	***P* < .001**
＜50	6557 (30.18)	141 (25.25)	32 (25.53)	105 (28.35)	113 (23.14)
≥50	15 169 (69.82)	418 (74.75)	92 (74.47)	264 (71.65)	378 (76.86)
Race		***P* < .001**	*P* = .580	*P* = .079	***P* < .001**
White	15 573 (71.68)	381 (68.14)	91 (73.05)	255 (69.02)	321 (65.29)
Black	4465 (20.55)	149 (26.61)	27 (21.99)	92 (24.93)	132 (26.86)
Other^a^	1560 (7.18)	29 (5.25)	6 (4.96)	22 (6.04)	39 (7.84)
Unknown	128 (0.59)	0 (0.00)	0 (0.00)	0 (0.00)	0 (0.00)
Marital status		***P* < .001**	***P* < .001**	***P* < .001**	***P* < .001**
Unmarried^b^	8634 (39.74)	294 (52.54)	69 (55.32)	193 (52.23)	280 (56.86)
Married	11 941 (54.96)	242 (43.22)	51 (41.13)	156 (42.26)	185 (37.65)
Unknown	1151 (5.30)	24 (4.24)	4 (3.55)	20 (5.51)	27 (5.49)
Grade		***P* < .001**	***P* < .001**	***P* < .001**	***P* < .001**
Well	354 (1.63)	10 (1.86)	2 (1.42)	2 (0.52)	6 (1.18)
Moderately	3498 (16.10)	99 (17.63)	21 (17.02)	61 (16.54)	58 (11.76)
Poorly	16 938 (77.96)	375 (67.12)	77 (62.41)	262 (71.13)	368 (74.71)
Undifferentiated	154 (0.71)	9 (1.53)	4 (2.84)	5 (1.31)	6 (1.18)
Unknown	782 (3.60)	66 (11.86)	20 (16.31)	39 (10.50)	55 (11.18)
Laterality		***P* < .001**	***P* < .001**	***P* < .001**	***P* < .001**
Left	11 115 (51.16)	281 (50.34)	63 (51.06)	189 (51.18)	246 (50.00)
Right	10 607 (48.82)	271 (48.47)	59 (47.52)	177 (48.03)	244 (49.61)
Bilateral	4 (0.02)	7 (1.19)	2 (1.42)	3 (0.79)	2 (0.39)
Histology		***P* < .001**	***P* < .001**	***P* < .001**	***P* < .001**
IDC	19 311 (88.89)	467 (83.54)	103 (83.06)	330 (89.43)	427 (86.79)
MBC	664 (3.05)	10 (1.78)	5 (4.03)	3 (0.81)	26 (5.28)
MedBC	301 (1.39)	0 (0.00)	0 (0.00)	0 (0.00)	2 (0.41)
IDC‐ILC	270 (1.24)	19 (3.40)	4 (3.23)	9 (2.44)	4 (0.81)
ILC	230 (1.06)	31 (5.55)	3 (2.42)	10 (2.71)	4 (0.81)
IDC‐oth	630 (2.90)	9 (1.61)	3 (2.42)	5 (1.36)	10 (2.03)
AAC	208 (0.96)	2 (0.36)	0 (0.00)	1 (0.27)	0 (0.00)
IBC	112 (0.51)	21(3.76)	6 (4.84)	11 (2.98)	19 (3.86)
Tumor size(mm)		***P* < .001**	***P* < .001**	***P* < .001**	***P* < .001**
≤50	19 226 (88.49)	289 (51.70)	62 (50.00)	180 (48.78)	195 (39.63)
＞50	2353 (10.83)	214 (38.28)	49 (39.51)	162 (43.90)	261 (53.05)
Unknown	147 (0.68)	56 (10.02)	13 (10.48)	27 (7.32)	36 (7.32)
Node stage		***P* < .001**	***P* < .001**	***P* < .001**	***P* < .001**
Negative	14 165 (65.20)	116 (20.68)	22 (17.73)	72 (19.42)	101 (20.59)
Positive	7561 (34.80)	443 (79.32)	102 (82.27)	297 (80.58)	391 (79.41)
Radiotherapy		***P* < .001**	***P* < .001**	***P* < .001**	***P* < .001**
Yes	10 878 (50.07)	227 (40.68)	90 (72.34)	96 (25.98)	159 (32.35)
No/Unknown	10 847 (49.93)	332 (59.32)	34 (27.66)	273 (74.02)	333 (67.65)
Chemotherapy		*P* = .691	*P* = .872	*P* = .842	***P* = .016**
Yes	16 614 (76.47)	423 (75.76)	94 (75.89)	284 (76.90)	351 (71.43)
No/Unknown	5112 (23.53)	136 (24.24)	30 (24.11)	85 (23.10)	141 (28.57)

Bold indicates statistically significant value.

a Including American Indian/Alaskan native, Asian/Pacific Islander, and others—unspecified.

b Including divorced, separated, single (never married), unmarried, domestic partner, and widowed.

### 3.2 Association of patient characteristics with preferential sites of distant metastases

To further evaluate the potential patient characteristics associated with preferential distant metastatic sites, we first performed univariate regression analysis in metastatic patients. In general, race, marital status, and histologic types were considered as potential significant risk factors (Table 2). Multivariate analysis was then performed. After adjusting these factors, only histologic subtypes were independently correlated with distant metastasis pattern. As shown in Table 3, MBC predicted fewer bone (OR = 0.414) and liver (OR = 0.176) metastasis cases but more lung (OR = 3.307) metastasis cases. Our results also indicated a positive effect of ILC and NST‐ILC on bone (OR = 2.470 and 4.702, separately) metastasis and negative effect on lung metastasis (OR = 0.203 and 0.128, separately), in which the effects were more remarkable in pure ILC contrasted with NST‐ILC mixed tumor. Our results proved that histological subtypes were the indicators of preferential distant metastatic sites of patients with TNBC.

**Table 2:** Univariate analysis of predictive factors of preferential DMS

	Bone metastasis		Brain metastasis		Liver metastasis		Lung metastasis	
	OR (95% CI)	*P* value	OR (95% CI)	*P* value	OR (95% CI)	*P* value	OR (95% CI)	*P* value
Age at diagnosis, y								
<50	REF^a^	REF	REF	REF	REF	REF	REF	REF
≧50	1.000 (0.761‐1.315)	.998	0.983 (0.655‐1.475)	.934	0.864(0.660‐1.131)	.288	1.294(1.000‐1.676)	.050
Race								
White	REF	REF	REF	REF	REF	REF	REF	REF
Black	0.991 (0.754‐1.302)	.949	0.740 (0.483‐1.133)	.166	0.883 (0.662‐1.177)	.395	1.093 (0.833‐1.436)	.521
Other^b^	0.733 (0.456‐1.179)	.201	0.673 (0.300‐1.510)	.337	0.864 (0.514‐1.452)	.580	**1.648 (1.041‐2.608)**	**.033**
Marital Status								
Unmarried^c^	REF	REF	REF	REF	REF	REF	REF	REF
Married	1.119 (0.853‐1.467)	.418	0.852 (0.559‐1.3)	.458	0.782 (0.439‐1.391)	.402	**0.742 (0.580‐0.949)**	**.018**
Unknown	0.864 (0.48‐1.554)	.625	0.692 (0.236‐2.033)	.504	0.829 (0.463‐1.485)	.528	1.147 (0.653‐2.014)	.633
Primary Tumor Site								
Upper‐inner	REF	REF	REF	REF	REF	REF	REF	REF
Upper‐outer	0.872 (0.518‐1.469)	.607	0.725 (0.341‐1.541)	.403	0.946 (0.551‐1.624)	.840	1.154 (0.681‐1.956)	.595
Lower‐inner	1.088 (0.502‐2.358)	.831	0.621 (0.182‐2.123)	.448	0.880 (0.392‐1.974)	.756	1.331 (0.614‐2.884)	.468
Lower‐outer	0.659 (0.313‐1.389)	.273	0.549 (0.161‐1.867)	.337	0.908 (0.417‐1.977)	.809	1.664 (0.788‐3.515)	.182
Central portion	0.946 (0.463‐1.934)	.880	0.347 (0.091‐1.330)	.123	1.210 (0.582‐2.516)	.610	1.086 (0.528‐2.237)	.822
Axillary tail	4.079 (0.452‐36.769)	.210	—d	—	0.880 (0.150‐5.151)	.887	0.732 (0.125‐4.273)	.732
Others^e^	0.997 (0.603‐1.650)	.992	1.066 (0.524‐2.167)	.860	0.909 (0.540‐1.532)	.721	1.376 (0.827‐2.289)	.219
Grade								
Well	REF	REF	REF	REF	REF	REF	REF	REF
Moderately	0.473 (0.127‐1.762)	.264	1.029 (0.217‐4.887)	.972	3.743 (0.811‐17.278)	.091	0.769 (0.255‐2.323)	.642
Poorly	0.286 (0.079‐1.032)	.056	0.770 (0.169‐3.496)	.735	3.233 (0.718‐14.549)	.126	1.293 (0.444‐3.760)	.638
Undifferentiated	0.351 (0.070‐1.761)	.203	2.000 (0.306‐13.062)	.469	2.727 (0.436‐17.046)	.283	0.800 (0.185‐3.460)	.765
Unknown	0.324 (0.086‐1.215)	.095	1.302 (0.273‐6.216)	.741	2.697 (0.577‐12.613)	.208	1.056 (0.346‐3.216)	.924
Laterality								
Left	REF	REF	REF	REF	REF	REF	REF	REF
Right	1.072 (0.844‐1.360)	.569	0.998 (0.699‐1.425)	.989	1.010 (0.787‐1.296)	.939	1.123 (0.885‐1.426)	.339
Bilateral	3.135 (0.646‐15.221)	.156	1.948 (0.397‐9.562)	.411	0.944 (0.233‐3.814)	.935	0.345 (0.071‐1.676)	.187
Histology Type								
IDC	REF	REF	REF	REF	REF	REF	REF	REF
MBC	**0.414 (0.194‐0.884)**	**.023**	1.419 (0.534‐3.765)	.483	**0.176 (0.053‐0.583)**	**.004**	**4.719 (1.924‐11.576)**	**.001**
MedBC	—	—	—	—	—	—	—	—
IDC‐ILC	**2.470 (1.028‐5.934)**	**.043**	1.393 (0.471‐4.122)	.550	0.902 (0.397‐2.046)	.804	**0.198 (0.068‐0.579)**	**.003**
ILC	**4.702 (1.943‐11.381)**	**.001**	0.676 (0.204‐2.240)	.522	0.631 (0.301‐1.320)	.523	**0.132 (0.046‐0.376)**	**.001**
IDC‐oth	1.024 (0.391‐2.678)	.962	1.641 (0.464‐5.809)	.442	0.710 (0.248‐2.032)	.523	1.556 (0.587‐4.123)	.374
AAC	—	—	—	—	1.703 (0.106‐27.318)	0.707	—	—
IBC	1.194 (0.615‐2.319)	.600	1.483 (0.604‐3.639)	.390	0.721 (0.351‐1.477)	.721	1.149 (0.595‐2.219)	.678
Tumor size								
≦5 cm	REF	REF	REF	REF	REF	REF	REF	REF
>5 cm	0.833 (0.660‐1.051)	.123	0.957 (0.645‐1.418)	.825	1.135 (0.882‐1.460)	.326	1.226 (0.753‐1.826)	.463
Unknown	1.182 (0.795‐1.759)	.408	1.207 (0.640‐2.274)	.561	0.799 (0.503‐1.272)	.345	1.061 (0.691‐1.628)	.787
Lymph nodes								
Negative	REF	REF	REF	REF	REF	REF	REF	REF
Positive	1.063 (0.795‐1.421)	.681	1.282 (0.811‐2.029)	.288	1.175 (0.862‐1.600)	.307	1.065 (0.796‐1.425)	.672
Radiotherapy								
No/Unknown	REF	REF	REF	REF	REF	REF	REF	REF
Yes	**1.948 (1.506‐2.520)**	**.001**	**6.645 (4.477‐9.863)**	**.001**	**0.570 (0.433‐0.749)**	**.001**	0.878 (0.683‐1.129)	.310
Chemotherapy								
No/Unknown	REF	REF	REF	REF	REF	REF	REF	REF
Yes	0.938 (0.709‐1.240)	.652	0.974 (0.644‐1.474)	.902	1.053 (0.785‐1.412)	.732	1.018 (0.770‐1.346)	.901

Bold indicates statistically significant value.

a For calculation of OR value, a group of patients were defined as reference.

b Including American Indian/Alaskan native, Asian/Pacific Islander, and others—unspecified.

c Including divorced, separated, single (never married), unmarried, domestic partner, and widowed.

d The number of patients was not enough for further calculation.

e Including nipple and overlapping carcinoma

**Table 3:** Multivariate analysis of predictive factors of preferential DMS

	Bone metastasis		Brain metastasis		Liver metastasis		Lung metastasis	
	OR (95% CI)	*P* value	OR (95% CI)	*P* value	OR (95% CI)	*P* value	OR (95% CI)	*P* value
Race								
White	—d	—	—	—	—	—	REF^a^	REF
Black	—	—	—	—	—	—	0.907 (0.688‐1.195)	.486
Other^b^	—	—	—	—	—	—	1.603 (0.988‐2.602)	.056
Marital status			—	—	—	—		
Unmarried^c^	—	—	—	—	—	—	REF	REF
Married	—	—	—	—	—	—	0.81 (0.626‐1.049)	.110
Unknown	—	—	—	—	—	—	1.112 (0.626‐1.976)	.717
Histology type								
IDC	REF	REF	REF	REF	REF	REF	REF	REF
MBC	**0.414 (0.194‐0.884)**	**.023**	—	—	**0.176 (0.053‐0.583)**	**.004**	**3.307 (1.590‐6.879)**	**.001**
MedBC	—	—	—	—	—	—	—	—
IDC‐ILC	**2.470 (1.028‐5.934)**	**.043**	—	—	0.902 (0.397‐2.046)	.804	**0.203 (0.068‐0.602)**	**.004**
ILC	**4.702 (1.943‐11.381)**	**.001**	—	—	0.631 (0.301‐1.320)	.523	**0.128 (0.044‐0.366)**	**.001**
IDC‐oth	1.024 (0.391‐2.678)	.962	—	—	0.710 (0.248‐2.032)	.523	1.491 (0.551‐4.037)	.431
AAC	—	—	—	—	1.703 (0.106‐27.318)	.707	—	—
IBC	1.194 (0.615‐2.319)	.600	—	—	0.721 (0.351‐1.477)	.721	0.841 (0.426‐1.66)	.617

Bold indicates statistically significant value.

a For calculation of OR value, a group of patients were defined as reference.

b Including American Indian/Alaskan native, Asian/Pacific Islander, and others—unspecified.

c Including divorced, separated, single (never married), unmarried, domestic partner, and widowed.

d The number of patients was not enough for further calculation or not involved in multivariate analysis.

### 3.3 Effect of histologic heterogeneous on survival of patients with metastatic TNBC

As we have proved that histologic subtypes were the most important characteristic that influence the preferential distant metastatic sites of TNBC, prognostic significance of histologic subtypes were further explored. We first evaluated whether histologic subtypes among common distant metastatic sites could affect the survival time of patients with TNBC. The log‐rank analysis of DSS is shown in Table 4. Compared with IDC, lung metastasis in patients with ILC had significantly worse prognosis (HR = 3.787, 95% CI: 1.205‐11.905, *P* ***= ***.023). For patients with bone, brain and liver metastasis, no obvious differences were found among the groups. Regarding OS, the same results were found as DSS (HR = 3.569, 95% CI: 1.136‐11.212, *P* ***= ***.029) (Table S1).

**Table 4:** Analysis of DSS for histologic subtypes within specific DMS

	Bone metastasis		Brain metastasis		Liver metastasis		Lung metastasis	
	HR (95% CI)	*P* value	HR (95% CI)	*P* value	HR (95% CI)	*P* value	HR (95% CI)	*P* value
Histology type								
IDC	REF^a^	REF	**2.196** (**1.392‐3.465)**	**.001**	1.263 (0.948‐1.682)	.110	1.034 (0.802‐1.332)	.797
MBC	REF	REF	1.370 (0.192‐9.793)	.754	—b	—	1.937 (0.433‐8.654)	.387
MedBC	REF	REF	—	—	—	—	—	—
IDC‐ILC	REF	REF	0.856 (0.098‐7.470)	.888	0.201 (0.030‐2.091)	.201	—	—
ILC	REF	REF	2.876 (0.318‐25.984)	.347	1.098 (0.139‐8.688)	.929	**14.999** (**1.301‐172.883)**	**.030**
IDC‐oth	REF	REF	—	—	—	—	0.461 (0.076‐2.818)	.402
AAC	REF	REF	—	—	—	—	—	—
IBC	REF	REF	0.939 (0.11‐8.04)	.954	1.644 (0.393‐6.868)	.496	0.513 (0.144‐1.828)	.303

Bold indicates statistically significant value.

a For calculation of HR value, a group of patients were defined as reference.

b The number of patients was not enough for further calculation.

We also evaluated whether prognostic differences could be found among the four DMS of each histologic subtype. For DSS, IDC patients with brain metastasis showed significant worse prognosis when compared with bone metastasis. For patients with ILC, lung metastasis was a remarkable factor that indicated poorer prognosis in patients with TNBC (Table 5). Considering OS, the results were found in accordance with DSS, while ILC subtype also predicted poorer prognosis in brain metastasis (Table S2).

**Table 5:** Analysis of DSS for specific histologic subtypes with different DMS

	Bone		Brain		Liver		Lung	
	HR (95% CI)	*P* value	HR (95% CI)	*P* value	HR (95% CI)	*P* value	HR (95% CI)	*P* value
Histology type								
IDC	REF^a^	REF	**2.196 (1.392‐3.465)**	**.001**	1.263 (0.948‐1.682)	.110	1.034 (0.802‐1.332)	.797
MBC	REF	REF	1.370 (0.192‐9.793)	.754	—b	—	1.937 (0.433‐8.654)	.387
MedBC	REF	REF	—	—	—	—	—	—
IDC‐ILC	REF	REF	0.856 (0.098‐7.470)	.888	0.201 (0.030‐2.091)	.201	—	—
ILC	REF	REF	2.876 (0.318‐25.984)	.347	1.098 (0.139‐8.688)	.929	**14.999** (**1.301‐172.883)**	**.030**
IDC‐oth	REF	REF	—	—	—	—	0.461 (0.076‐2.818)	.402
AAC	REF	REF	—	—	—	—	—	—
IBC	REF	REF	0.939 (0.11‐8.04)	.954	1.644 (0.393‐6.868)	.496	0.513 (0.144‐1.828)	.303

Bold indicates statistically significant value.

a For calculation of HR value, a group of patients were defined as reference.

b The number of patients was not enough for further calculation.

## 4 DISCUSSION

Triple‐negative breast cancer is one of the most aggressive subtypes with high frequency of distant metastasis which seriously impacts the prognosis of patients; hence, studies evaluating the correlation between patient characters and preferential distant metastatic sites are needed. In our large population–based cohort of cases diagnosed with TNBC, we eventually demonstrated that certain histological subtypes showed correlations to site‐specific metastasis patterns. Moreover, the site‐specific metastatic patients showed different prognosis among subtypes.

After reviewing published articles, research on breast cancer metastasis mainly focused on the gene level and numerous genes have been proved to play crucial roles on regulating tumor metastasis. A study on high‐resolution clonal mapping of multiorgan metastasis in TNBC revealed that tumors at different metastatic sites showed specific gene pattern. Lung, brain, liver, and multiorgan metastatic tumors have similar gene features.^13^ Another research found that the genomic mutations were originated from the primary tumor and maintained through metastatic spreading, of which TP53 mutation was a recurrent founding mutation in primary and metastatic tumors.^14^ We also reported genes like *NAMPT*, *SREBP1*, and *MTDH* could drive metastatic progression in TNBC.^15^, ^16^, ^17^ The alteration of genes cluster could not only influence malignant behaviors, but also transform pathomorphological features. Histologic subtypes of breast cancer were the consequence of genes alteration, which were greatly different in morphology, behavior, and mechanism. Previous studies also demonstrated that subtypes could own specific gene expression pattern, and it of great significance to evaluate heterogeneities among subtypes. For instance, GATA3 is not only detected in metaplastic and lobular breast cancer of TNBC, but also is highly expressed in other subtypes.^18^ Moreover, it has been reported that medullary carcinoma had the lowest tissue levels of estrogen and progesterone receptors while mucinous carcinoma had the highest percentages of positive estrogen and progesterone receptor levels.^19^ In our study, most patients with TNBC were invasive ductal carcinoma not otherwise specified. The remaining 10%‐25% of patients comprise medullary carcinoma, metaplastic carcinoma, neuroendocrine carcinoma, adenoid cystic carcinoma, invasive lobular carcinoma, apocrine carcinoma, mixed lobular‐ductal carcinoma, and inflammation breast cancer.^20^, ^21^, ^22^

TNBC was supposed to have the preference to metastasis to brain and visceral organs, such as lung and liver, rather than bone compared to other breast cancer molecular subtypes.^23^ On further research of predictive factors of preferential DMS in patients with TNBC, we concluded that histologic subtypes were the only independent factor. Our results showed patients with MBC showed fewer risks of bone and liver metastasis but more lung metastasis compared with IDC. An interesting study published recently reported the activity of HER2 pathway was significantly lower in MBC samples than in IDC samples although all patients were clinically categorized as negative for HER2 amplification.^24^ Based on the above study, it has been reported that the activation of HER‐2/CXCR4/ Akt signaling pathway in primary breast tumors could contribute to the formation of bone metastases in breast cancer,^25^ and HR‐negative/HER2‐positive subtype patients had a considerably high proportion of liver metastasis,^12^ which might account for fewer bone and liver metastasis of MBC. Considering the correlation between HER‐2 and lung metastasis, we found that HER‐2 inactivation contributed to lung metastasis,^26^ and the inactivation of HER‐2 pathway in MBC could result in more lung‐specific distant metastasis. For patients with ILC or IDC‐ILC, a totally opposite result was found, in which more bone and fewer lung metastasis compared with IDC patients, and were in accordance with published articles.^27^, ^28^ Based on published articles, we found CDH1 was one of key markers that could distinguish ILC from IDC.^29^ The loss of expression is observed in the majority of lobular breast carcinomas, CDH1 integrity is impaired.^30^ On the contrary, the expression is unaffected in ductal breast carcinomas.^31^ In some articles, roles of CDH1 on distant metastasis of breast cancer have been illustrated. For instance, Maroni et al reported that CDH1 were expressed in bone metastasis but not in primary breast carcinoma, which playing a pivotal role in bone metastasis colonization.^32^ Another article also demonstrated crucial roles of CDH1 on promoting bone metastasis.^33^ The loss of expression of the cell‐cell adhesion molecule CDH1 in ILC might account in part for the different metastatic patterns observed in these types of tumors. Based on our results and previous studies, histologic subtypes of breast cancer owned specific malignant behaviors and molecular mechanisms.

Another aspect of our research was to evaluate prognoses among patients with TNBC with different histologic subtypes and different distant metastasis sites. Based on previous studies, it has been reported that histologic subtypes of TNBC showed significantly various prognoses, which adenoid cystic carcinoma and medullary breast carcinoma owned the longest overall survival, and the prognosis of ILC was worst.^34^ For metastatic patients, there was no study that systematically assessed prognosis among subtypes. In our study, we first evaluated prognosis of different subtypes within common distant metastasis sites. When compared with IDC, only ILC with lung metastasis showed significant differences for both DSS and OS, which significantly correlated with poorer prognosis. Despite patients with ILC were unlikely to develop lung metastasis, it caused much more deaths among metastatic patients compared with IDC. Mechanism of poorer prognosis for patients with ILC with lung metastasis have not been reported before. Based on previous studies, ILC is more common in older age, tends to be multicentric, often present as larger tumors with ill‐defined margins and have a unique metastatic pattern,^28^, ^35^, ^36^ which could partly account for the prognoses. Invasive lobular breast cancer is the second most common histologic type of invasive breast cancer and accounts for 5%‐10% of all breast cancer cases,^37^, ^38^ it of great value to further evaluating mechanism and clinical significance of our results. It has been proved that TNBC with various distant metastasis sites showed different prognoses. We further analyzing prognoses difference of common subtypes with various distant metastatic sites. Compared with bone metastasis, we found occurrence of brain metastasis of IDC indicated remarkable poorer prognoses, which were in accordance with previous results.^23^ However, it is surprising that patients with ILC with lung metastasis showed the worst prognoses instead brain metastasis. Based on previously reports, breast cancer patients with lung and bone metastasis owned comparative longer prognoses than brain and liver metastases.^23^ Conversely, we found that patients with triple‐negative ILC with lung metastasis had worst prognosis followed by brain metastasis, the follow‐up and medical examination of patients with ILC should pay more attentions. The clear mechanism of ILC has not been reported before, which might result from the unique gene expression pattern. It is of great value for furthering exploring the difference between IDC and ILC, which might reveal a cluster of key genes in breast cancer.

**Figure 1:**
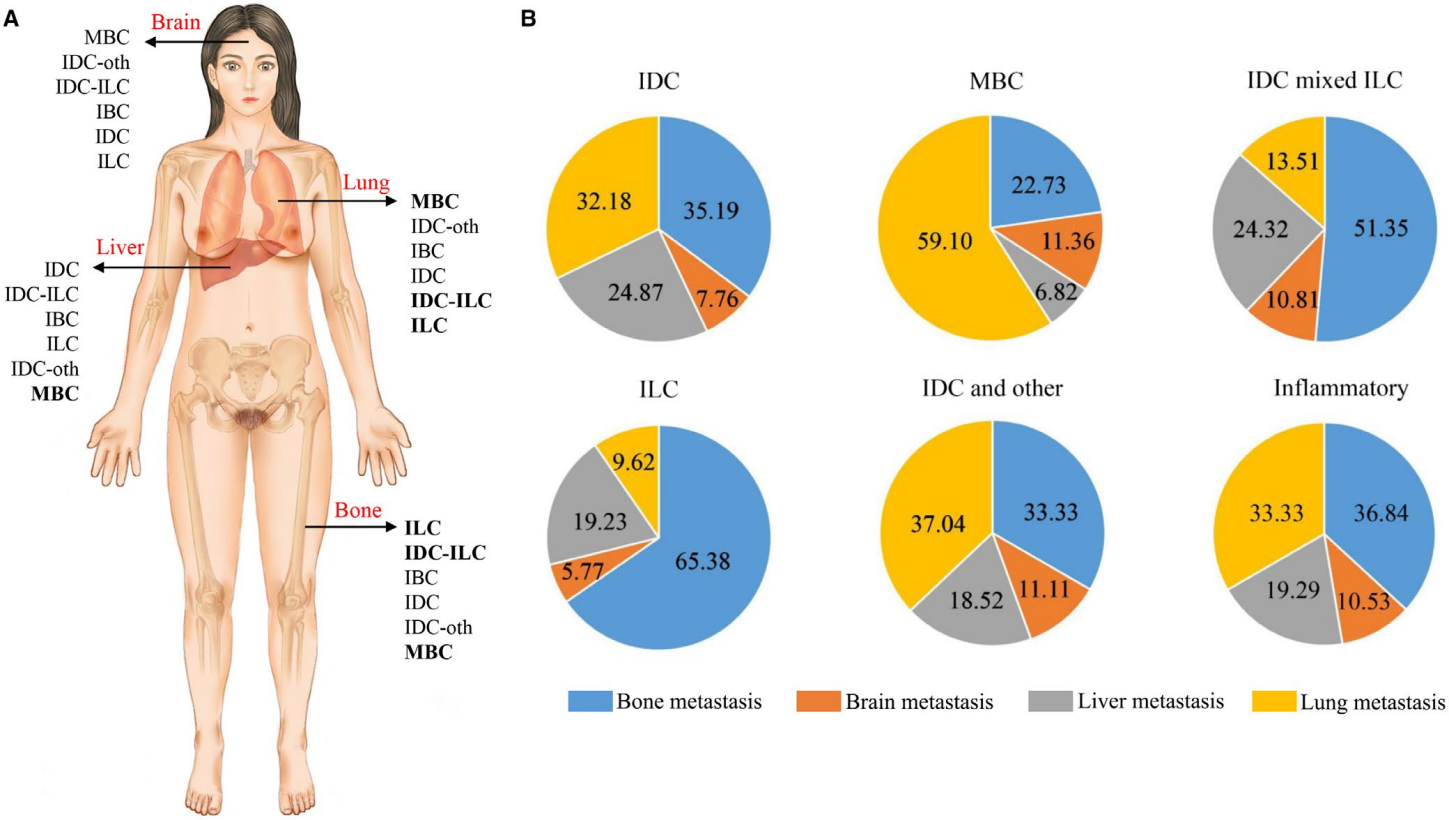
Preferential distant organ metastasis patterns of histologic subtypes. A, Human body schematic diagram presented the preferential distant metastatic sites of histologic subtypes. The orders of histologic subtypes in each organ were sorted from high to low preference and subtypes with significance were bolded. MedBC and AAC were not involved for lack of metastasized patients. B, Pie graphs that represented the proportion of site‐specific distant metastases of each histologic subtype

Figure 1 shows human body schematic diagram and pie graphs that represents the proportion of distant metastasis of histologic subtypes. Compared with patients with IDC, with 35.19% bone, 7.76% brain, 24.87% liver, and 32.18% lung metastasis, the patients with MBC showed remarkable decrease in bone and liver and increase in lung metastasis. For ILC mixed ILC and ILC subtypes, more patients trend to more bone metastasis and less lung metastasis. For IDC and other histologic subtypes of breast cancer, no obvious difference was found.

There are several limitations of this study. First, retrospective studies are inherently biased. Second, the SEER database only included four specific sites of distant metastases at the initial diagnosis, and we could not obtain further details concerning the time of secondary metastasis. Third, the number of patients for certain subtypes were not enough to make significant results. In addition, we only included clinical characteristics in this study, which were obtained from SEER database, while other factors including gene expression should also be taken into consideration in further studies.

## 5 CONCLUSION

In summary, this study proved that certain histologic subtypes of breast cancer are associated with metastatic behavior regarding the sites of distant metastasis and prognosis, of which patients with MBC and ILC should pay more attentions. This knowledge may help to further understand the mechanism of breast cancer metastasis and to monitor the prognosis of patients with TNBC.

## CONFLICT OF INTEREST

The authors declare no conflict of interest.

## AUTHOR CONTRIBUTIONS

Yaming Li analyzed the data and wrote the paper; Jie Li and Qifeng Yang designed the study; Peng Su and Xiaoyan Li provided the technical support for data analysis; Yifei Wang and Hanwen Zhang wrote the paper and drawn the picture; Yiran Liang, Ning Zhang, and Xiaojin Song collected and analyzed the data.
