# Corrigendum

**DOI:** 10.1002/cam4.3415

**Published:** 2020-08-28

In the article by Li et al.,^1^ the published version of Table 4 is incorrect. Its content is identical with Table 5

The correct version of Table 4 is displayed below:

**Table 4:** Analysis of DSS for histologic subtypes within specific DMS

	Bone metastasis		Brain metastasis		Liver metastasis		Lung metastasis	
	HR (95% CI)	*P* value	HR (95% CI)	*P* value	HR (95% CI)	*P* value	HR (95% CI)	*P* value
Histology Type								
IDC	REF^a^	‐	REF	‐	REF	‐	REF	‐
MBC	1.06(0.525‐2.14)	.87	0.894 (0.318‐2.515)	.832	0.949 (0.236‐3.823)	.942	1.381 (0.895‐2.133)	.145
Medullary	‐^b^	‐	‐	‐	‐	‐	2.812 (0.696‐11.357)	.147
IDC and LC	1.202 (0.703‐2.054)	.502	0.627 (0.197‐1.99)	.428	0.804 (0.397‐1.629)	.545	0.72 (0.23‐2.252)	.572
LC	0.693 (0.43‐1.117)	.132	1.438 (0.351‐5.884)	.613	1.549 (0.764‐3.139)	.225	**3.787 (1.205‐11.905)**	**.023**
IDC and other	1.289 (0.574‐2.893)	.539	0.528 (0.073‐3.801)	.526	1.017 (0.377‐2.738)	.974	0.534 (0.219‐1.298)	.166
Apocrine	1.619 (0.403‐6.51)	.497	‐	‐	3.772(0.525‐27.092)	.187	‐	‐
Inflammatory	1.425 (0.861‐2.358)	.169	1.282 (0.558‐2.945)	.558	1.53(0.783‐2.989)	.213	1.11(0.636‐1.936)	.714

Bold indicates statistically significant value.

^a^ For calculation of HR value, a group of patients were defined as reference.

^b^ The number of patients was not enough for further calculation.

The authors apologize for this error.

## References

[1] Li Y , Su P , Wang Y , et al. Impact of histotypes on preferential organ‐specific metastasis in triple‐negative breast cancer. Cancer Med. 2020;9:872–881. 10.1002/cam4.2759 31814295 PMC6997059

