# Supplementary material for: Impact of histotypes on preferential organ‐specific metastasis in triple‐negative breast cancer
Source: Cancer Med. 2019 Dec 9;9(3):872–81. doi: 10.1002/cam4.2759 (PMC6997059; doi:10.1002/cam4.2759)
Supplement: Supplementary file 1 [file CAM4-9-872-s001.docx]

**Table S1: Analysis of OS for histologic subtypes within specific DMS**

|  | **Bone metastasis** | | **Brain metastasis** | | **Liver metastasis** | | **Lung metastasis** | |
| --- | --- | --- | --- | --- | --- | --- | --- | --- |
|  | **HR (95% CI)** | **P value** | **HR (95% CI)** | **P value** | **HR (95% CI)** | **P value** | **HR (95% CI)** | **P value** |
| **Histology Type** |  |  |  |  |  |  |  |  |
| IDC | REF^1^ | REF | REF | REF | REF | REF | REF | REF |
| MBC | 0.977(0.484-1.97) | 0.947 | 0.878(0.312-2.468) | 0.805 | 0.889(0.221-3.577) | 0.868 | 1.311(0.85-2.023) | 0.220 |
| MedBC | -^2^ | - | - | - | - | - | 2.668(0.661-10.769) | 0.168 |
| IDC-ILC | 1.174(0.7-1.971) | 0.543 | 0.613(0.193-1.947) | 0.407 | 0.756(0.373-1.53) | 0.436 | 0.691(0.221-2.162) | 0.526 |
| ILC | 0.68(0.427-1.081) | 0.103 | 2.092(0.657-6.666) | 0.212 | 1.444(0.713-2.924) | 0.308 | **3.569(1.136-11.212)** | **0.029** |
| IDC-oth | 1.182(0.527-2.652) | 0.685 | 0.515(0.072-3.707) | 0.51 | 0.963(0.358-2.591) | 0.94 | 0.513(0.211-1.247) | 0.141 |
| AAC | 1.482(0.369-5.955) | 0.579 | - | - | 3.423(0.477-24.558) | 0.221 | - | - |
| IBC | 1.376(0.844-2.243) | 0.200 | 1.249(0.544-2.866) | 0.600 | 1.429(0.732-2.789) | 0.295 | 1.134(0.663-1.938) | 0.647 |

1. For calculation of HR value, a group of patients were defined as reference.
2. The number of patients was not enough for further calculation.

**Table S2: Analysis of OS for specific histologic subtypes with different DMS**

|  | **Bone** | | **Brain** | | **Liver** | | **Lung** | |
| --- | --- | --- | --- | --- | --- | --- | --- | --- |
|  | **HR (95% CI)** | **P value** | **HR (95% CI)** | **P value** | **HR (95% CI)** | **P value** | **HR (95% CI)** | **P value** |
| **Histology Type** |  |  |  |  |  |  |  |  |
| IDC | REF^1^ | REF | **1.897(1.208-2.980)** | **0.005** | 1.212(0.923-1.591) | 0.166 | 0.963(0.756-1.227) | 0.963 |
| MBC | REF | REF | 1.370(0.192-9.793) | 0.754 | -^2^ | - | 1.937(0.433-8.654) | 0.387 |
| MedBC | REF | REF | - | - | - | - | - | - |
| IDC-ILC | REF | REF | 0.707(0.086-5.833) | 0.766 | 0.323(0.058-1.803) | 0.362 | - | - |
| ILC | REF | REF | **5.672(1.028-31.289)** | **0.046** | 1.023(0.131-8.008) | 0.983 | **17.694(1.500-208.733)** | **0.022** |
| IDC-oth | REF | REF | - | - | - | - | 0.461(0.076-2.818) | 0.402 |
| AAC | REF | REF | - | - | - | - | - | - |
| IBC | REF | REF | 0.742(0.089-6.183) | 0.783 | 1.267(0.316-6.5.079) | 0.738 | 0.564(0.178-1.786) | 0.330 |

1. For calculation of HR value, a group of patients were defined as reference.
2. The number of patients was not enough for further calculation.
